# Vertical Aerosol Distribution and Mesospheric Clouds From ExoMars UVIS

**DOI:** 10.1029/2021JE007065

**Published:** 2022-04-27

**Authors:** Paul M. Streeter, Graham Sellers, Michael J. Wolff, Jonathon P. Mason, Manish R. Patel, Stephen R. Lewis, James A. Holmes, Frank Daerden, Ian R. Thomas, Bojan Ristic, Yannick Willame, Cédric Depiesse, Ann Carine Vandaele, Giancarlo Bellucci, José Juan López‐Moreno

**Affiliations:** ^1^ School of Physical Sciences The Open University Milton Keynes UK; ^2^ Space Science Institute Boulder CO USA; ^3^ Space Science and Technology Department Science and Technology Facilities Council Rutherford Appleton Laboratory Oxfordshire UK; ^4^ Royal Belgian Institute for Space Aeronomy (IASB‐BIRA) Brussels Belgium; ^5^ Istituto di Astrofisica e Planetologia Spaziali (IAPS/INAF) Rome Italy; ^6^ Instituto de Astrofìsica de Andalucía (IAA) Consejo Superior de Investigaciones Científicas (CSIC) Granada Spain

**Keywords:** ExoMars TGO, clouds, NOMAD, UVIS, Mars atmosphere, data assimilation

## Abstract

The vertical opacity structure of the martian atmosphere is important for understanding the distribution of ice (water and carbon dioxide) and dust. We present a new data set of extinction opacity profiles from the NOMAD/UVIS spectrometer aboard the ExoMars Trace Gas Orbiter, covering one and a half Mars Years (MY) including the MY 34 Global Dust Storm and several regional dust storms. We discuss specific mesospheric cloud features and compare with existing literature and a Mars Global Climate Model (MGCM) run with data assimilation. Mesospheric opacity features, interpreted to be water ice, were present during the global and regional dust events and correlate with an elevated hygropause in the MGCM, providing evidence that regional dust storms can boost transport of vapor to mesospheric altitudes (with potential implications for atmospheric escape). The season of the dust storms also had an apparent impact on the resulting lifetime of the cloud features, with events earlier in the dusty season correlating with longer‐lasting mesospheric cloud layers. Mesospheric opacity features were also present during the dusty season even in the absence of regional dust storms, and interpreted to be water ice based on previous literature. The assimilated MGCM temperature structure agreed well with the UVIS opacities, but the MGCM opacity field struggled to reproduce mesospheric ice features, suggesting a need for further development of water ice parameterizations. The UVIS opacity data set offers opportunities for further research into the vertical aerosol structure of the martian atmosphere, and for validation of how this is represented in numerical models.

## Key Points

- Mesospheric water ice clouds were present in ExoMars/UVIS opacity profiles during a global and three regional dust storms
- Mesospheric water ice correlated with elevated hygropause, showing storms boost vertical water transport with hydrogen escape implications
- Perihelion season mesospheric water ice was also present even in the absence of global/regional dust storm activity

## 1 Introduction

Suspended atmospheric aerosols are key components of the martian atmosphere, and their vertical distribution has long been a subject of investigation with orbital observations and modeling. The aerosols found in Mars' atmosphere are mineral dust, water ice, and CO_2_ ice, and each have distinct spatiotemporal distributions and radiative effects. Early analytical calculations based on estimated dust sedimentation and diffusion rates alongside orbital observations of a Global Dust Storm (GDS) led to the development of a standard dust profile often used in numerical modeling work, where dust opacity is considered to be constant in a well‐mixed bottom layer of the atmosphere before decreasing monotonically at higher altitudes (Conrath, 1974). More recent observations from instruments able to examine the atmospheric limb have revealed a more complex dust vertical structure, including the presence of local dust maxima (detached dust layers) at lower altitudes, particularly during the less dusty aphelion season (e.g., Heavens et al., 2011b; Guzewich, Talaat, et al., 2013), and large plume‐like convective dust structures during GDS (Heavens et al., 2019). Such non‐monotonic vertical dust structure is linked to still‐debated transport processes involving slope flows and local convective activity (e.g., Daerden et al., 2015; C.Wang et al., 2018; Heavens et al., 2015; Spiga et al., 2013), and its representation in Mars Global Climate Models (MGCM) has been shown to have noticeable effects on the thermal structure and circulation (Guzewich, Toigo, et al., 2013). In general, dust appears to be confined below around 20 km during the aphelion season and below 30–50 km during the more active perihelion season (Smith et al., 2013). The presence of dust is also linked to hydrogen escape from the martian atmosphere, which has been shown to be enhanced by atmospheric heating and boosted by vertical transport of water from GDS (Chaffin et al., 2014; Heavens et al., 2018) and regional dust storms (J. Holmes et al., 2021; Chaffin et al., 2021).

The distribution of water and CO_2_ condensates is tied to the seasonal behavior of their relevant cloud formations. In general, the formation of water ice clouds requires sufficient presence of water, low enough temperatures for condensation, and cloud condensation nuclei (CCN) (Michelangeli et al., 1993). The aphelion cloud belt (ACB) is an annual water ice feature visible straddling the tropics during the aphelion season due to the lower atmospheric temperatures, with peak opacity values at around *L*_S_ = 100° (e.g., Clancy et al., 1996; Mateshvili et al., 2009; Smith, 2008; Smith, 2009), with altitudes of around 17–45 km depending on latitude ‐ tending to be higher in the northern tropics than the southern (Smith et al., 2013). ACB opacities have been observed to be higher at 1700 local time than at 1400 local time (Smith, 2009), and indeed show a high local time variability due to the close dependence of water ice formation on atmospheric temperature, with ice opacity being highest from local midnight to local dawn and lowest at local noon (Giuranna et al., 2021). Another annual water ice feature is the presence of polar hood clouds around both poles from late hemispheric summer to early spring (H. Wang & Ingersoll, 2002), linked to cooler temperatures and the availability of CCN from circumpolar baroclinic dust storms (Cantor et al., 2010). Both polar hood cloud features range from approximately 10‐40 km in altitude, with opacity being highly dependent on atmospheric temperature changes (Benson et al., 2010, 2011). However, Vincendon et al. (2011) identified water ice clouds at mesospheric (here defined as >40 km) altitudes of up to 80 km during Mars' aphelion season. And recently, Clancy et al. (2019) have detected mesospheric (here defined as >40 km) water ice clouds during Mars' perihelion season between 50 and 90 km altitudes and across the whole of their study's observable range of 50° S‐50° N, with a minimum toward lower latitudes.

Lastly, Mars' low atmospheric temperatures enable the characteristically martian phenomenon of CO_2_ ice clouds, first definitively detected by Formisano et al. (2006). These features have been detected at high altitudes above the tropics and subtropics, generally in the 65–100 km altitude range in the aphelion season when the atmospheric temperature is particularly low (e.g., Aoki et al., 2018; Clancy et al., 2019; Määttänen et al., 2010; McConnochie et al., 2010; Montmessin et al., 2006); MGCMs are currently unable to replicate these low temperatures, which may be the effect of dynamical phenomena such as gravity waves and thermal tides (e.g., González‐Galindo et al., 2011; Spiga et al., 2012). Such phenomena may have to do with the apparent ephemerality of CO_2_ ice clouds, which may have lifetimes as low as minutes (Listowski et al., 2014); indeed, while there are now many reported detections, CO_2_ ice presence is sparse in retrievals, for example, only appearing in less than 1% of Spectroscopy for the Investigation of the Characteristics of the Atmosphere of Mars (SPICAM) mesospheric retrievals (Montmessin et al., 2006). Likewise, stellar occultations from the Imaging Ultraviolet Spectrograph (IUVS) aboard the Mars Atmosphere and Volatile Evolution (MAVEN) spacecraft showed high‐altitude (>90 km) CO_2_ clouds in only 2 of 32 supersaturated CO_2_ profiles, out of a total of 309 stellar occultation profiles (Jiang et al., 2019). Longer‐lasting and optically thicker low‐altitude (<25 km) CO_2_ ice clouds have also been detected in Mars' polar night (Hayne et al., 2012).

The spectrometer instruments aboard the ExoMars Trace Gas Orbiter (TGO), launched in 2016, have already helped contribute to the understanding of martian atmospheric aerosol distribution. Nadir and Occultation for MArs Discovery (NOMAD) (Vandaele et al., 2015) and Atmospheric Chemistry Suite (ACS) (Korablev et al., 2015) are both spectrometer suites aboard TGO and have been observing the martian atmosphere since 2018 in both nadir and solar occultation modes. Aoki et al. (2019) used NOMAD to investigate the behaviour of water vapour during the 2018 GDS, finding that water vapour presence in the mesosphere (40–100 km) increased substantially during the storm, particularly between 60° S and 60° N in the growth stages (*L*_S_ = 195–220°) and at higher latitudes in the decay stages (*L*_S_ = 220–260°); the later Mars Year 34 (MY 34) regional dust storm (RDS) had similar effects. Also with NOMAD, Liuzzi et al. (2020) examined the behaviour of water ice clouds during the storm, finding that the storm induced a high‐altitude mesospheric water ice layer which rose from 45 to 80 km rapidly after the GDS inception, as well as greater water ice abundance at local dusk than at dawn. Using ACS, A. A. Fedorova et al. (2020) found increased high‐altitude water vapour, ice, and water saturation during the MY 34 GDS period and the regional dust storm. And again with ACS, Stcherbinine et al. (2020) observed a shift in maximum water ice cloud altitudes from 60 km to above 90 km during the GDS.

Total extinction opacity profiles of the martian atmosphere from other instruments have previously provided insights into the vertical aerosol distribution. Solar occultations from the Phobos 2 spacecraft provided aerosol profiles for northern spring at the equator, allowing identification of water ice clouds from thermal considerations (Chassefière et al., 1992). Solar occultations from SPICAM have been used to obtain aerosol extinction profiles in the 1–1.7 *μ*m range between 10 and 60 km, enabling constraints on top of the haze layer at 40 km and detection of mesospheric clouds between 50 and 60 km (Fedorova et al., 2009). SPICAM's UV channel has also been used to investigate aerosol opacity profiles via solar occultations, showing the seasonal dust behaviour of the atmosphere and the presence of high‐altitude detached layers during the 2007 GDS (Määttänen et al., 2013).

This paper describes an opacity profile data set derived from solar occultations performed by the UV and visible spectrometer (UVIS) (M. R. Patel et al., 2017) on the NOMAD instrument, extending from *L*_S_ = 180° in MY 34 to the end of MY 35. This period covers one and a half martian years, including the MY 34 GDS, the MY 34 regional dust storm (which began at around *L*_S_ = 320°), and the non‐GDS year of MY 35. The coverage of this data set therefore offers the opportunity for investigation of the extreme conditions of an equinoctial GDS as well as the more average conditions of a non‐GDS martian year. This data set does not contain retrievals of specific dust and ice opacities or properties, instead providing total extinction opacities which do not distinguish between aerosol types. The advantage of this approach is that it does not introduce bias or errors from the specifics of a full aerosol retrieval process, but provides an accurate measured of overall extinction which can be used to investigate total aerosol structure in the atmosphere. It is also technically simpler, enabling the UVIS opacity profile data set to be updated almost in real time from UVIS solar occultations, providing a quick and comprehensive overview of the aerosol structure of the martian atmosphere. The structure of this paper is as follows. We first describe the process by which the UVIS opacities are obtained, and the MGCM with data assimilation we later compare the UVIS data set to. We then examine specific cloud opacity features in the data set and compare them to the MGCM with data assimilation, and discuss these results in the context of previous work. The intention of this data set is to provide the community with an opacity‐based data set upon which further investigations, such as particle size and composition, can be performed.

## 2 Methods

### 2.1 UVIS Opacity Calculation

The opacity profiles used in this study are taken from a larger retrieval data set derived from UVIS occultations designed to extract other spectral signatures from the data in addition to total aerosol content. The complete retrieval process is described in detail in M. R. Patel, Sellers, et al. (2021), and briefly summarized here for convenience.

**Figure 1:**
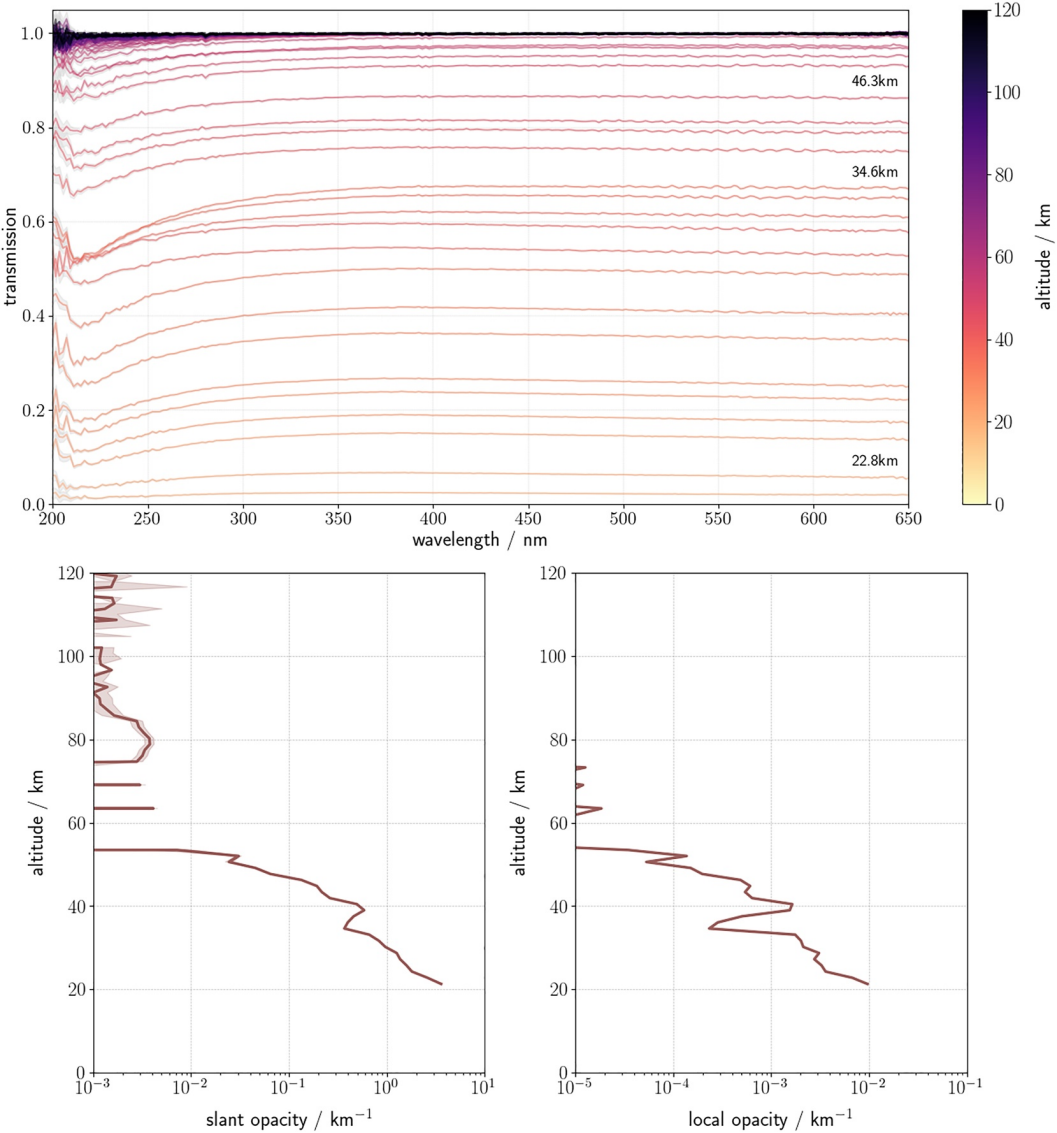
The measured transmission spectra from an example occultation observed on 2018–10–30 at 19:32:49 (UTC) (top). Aerosol slant opacities at 320 nm are extracted from these transmissions using the Beer‐Lambert law (bottom left) and an onion‐peeling based vertical inversion procedure is conducted on the obtained slant opacities to produce local opacity vertical profiles (bottom right).

NOMAD solar occultations profiles are used covering the period from MY 34 *L*_S_ = 160° to MY 35 *L*_S_ = 360°. Solar occultation profiles are self‐calibrated in that the measurements are expressed as a transmission value by considering the average of measurements made above the altitude where UVIS can detect the atmosphere (∼120 km) as the reference spectrum. Measurements made below this altitude are then expressed as a transmission using this occultation‐specific reference. Transmissions <1% are not considered in this study due to lack of signal. The transmission spectra observed by the instrument at successive tangent altitudes above the surface (Figure 1, top) are converted into slant opacities by using the Beer‐Lambert Law. The slant opacity is calculated over the wavelengths 320–360 nm, chosen in order to avoid the Hartley band of ozone absorption (centered at 255 nm) and the poor performance of the detector at shorter wavelengths (M. R. Patel, Sellers, et al., 2021). The slant density profile (Figure 1, bottom left) is converted to an associated local density profile following a variant of an established onion peeling process (Auvinen et al., 2002; Quémerais et al., 2006; Rodgers, 2000). Assuming a spherically symmetric atmosphere in the plane of the observations, the line‐of‐sight path lengths through each of these atmospheric layers are then calculated geometrically to yield opacity profiles (Figure 1, bottom right). Uncertainties on the vertical profiles are calculated via an analogous method of inversion; due to inherent uncertainties in the observed transmissions, the resultant detection limit of opacity is defined as ∼10^−5^.

### 2.2 MGCM and Assimilation Details

The MGCM used to compare against the UVIS data is the result of a collaboration between the Laboratoire de Météorologie Dynamique, the University of Oxford, the Open University, and the Instituto de Astrofísica de Andalucía (Forget et al., 1999). The MGCM is a 4D numerical model of the martian atmosphere which solves the primitive equations of fluid dynamics with a spectral dynamical core; in the vertical dimension, it uses a finite‐difference representation; and tracer advection is performed using a semi‐Lagrangian scheme (Lewis et al., 2007). Dust and water tracers are advected with a log‐normal size distribution, as part of a two‐moment scheme, with the total optical depth of dust at each column of the atmosphere (column dust optical depth; CDOD) being scaled to match assimilated observations of CDOD (see below) (Madeleine et al., 2011; P. M. Streeter et al., 2020); however, the vertical distribution of dust is not prescribed, and is allowed to evolve freely with the MGCM. The representation of dust in the MGCM is radiatively active, meaning that dust interacts with shortwave and longwave radiation and can influence atmospheric temperatures; the specific radiative properties of dust used for the MGCM are from Wolff et al. (2006); Wolff et al. (2009), as derived from observations. The MGCM uses a radiative transfer scheme which is robust even at the very high CDOD values observed for the MY 34 GDS, to within ∼10% error (Toon et al., 1989; P. M. Streeter et al., 2020). A water cycle is also included as part of the MGCM and includes water ice cloud microphysics and radiatively active water ice (Navarro et al., 2014); this was used for the runs shown in this study.

The MGCM was run together with a modified version of the Analysis Correction data assimilation scheme (Lorenc et al., 1991), adapted for the case of the martian atmosphere (Lewis et al., 1997, 2007). Retrieved temperature profiles from Mars Climate Sounder (MCS) aboard the Mars Reconnaissance Orbiter (MRO) were assimilated following the technique previously used with this MGCM and assimilation scheme for Thermal Emission Spectrometer (TES) (Lewis et al., 2007; J. A. Holmes et al., 2018) and MCS (Holmes, Lewis, Patel, et al., 2019; Steele et al., 2014) temperatures, while CDOD derived from MCS limb measurements was assimilated to constrain MGCM dust columns as described above (Lewis et al., 2007). The MGCM was run with data assimilation, which combines model and observational inputs for the purpose of creating the best possible estimate of the state of the atmosphere. The particular data assimilation scheme used for the MGCM is a version of the UK Met Office's Analysis Correction scheme (Lorenc et al., 1991), modified for the specific conditions of Mars' atmosphere (Lewis et al., 1997, 2007). For this study, the observations assimilated into the MGCM were retrieved temperature profiles and derived CDOD from the Mars Reconnaissance Orbiter (MRO)'s Mars Climate Sounder (MCS). The temperature profiles were assimilated following the technique described by Holmes, Lewis, Patel, et al. (2019); Steele et al. (2014) and previously used with the same MGCM and assimilation scheme. Assimilated MCS temperatures were filtered to avoid falling below the CO_2_ frost point, which can cause runaway CO_2_ condensation in the MGCM. The derived CDOD products were assimilated to constrain MGCM CDOD as described in the previous paragraph. MCS is a limb‐pointing instrument and is therefore unable to directly retrieve CDOD; however, from limb retrievals of dust opacity it is possible to extrapolate a CDOD value. MCS retrieves reports dust opacities at infrared wavelengths (21.6 *μ*m); however, the MGCM performs its dust radiative transfer at visible wavelengths (670 nm). Derived MCS CDOD values were therefore scaled by a conversion factor of 7.3 prior to assimilation (Kleinböhl et al., 2011). Prior to assimilation of CDOD, dayside equatorial CDOD values were filtered out in order to prevent possibly spuriously high CDOD values which have been known to occur (Montabone et al., 2015); this filtering was suspended for the MY 34 GDS period (Montabone et al., 2020). The retrieval version used for assimilation of temperature and dust was v5.2, except for during the period of the MY 34 GDS, when v5.3.2 was used for dust. The MGCM and assimilation process were the same as previously used for the OpenMARS data set, which currently extends to MY 32 (Holmes, Lewis, Patel, et al., 2019).

Temperature profiles from MCS are reported for altitudes of up to approximately 85 km and have an intrinsic vertical resolution of approximately 5 km (Kleinböhl et al., 2009). The specifics of MRO's orbit results in MCS observations having two (approximately) fixed local times, specifically 0300 and 1500 at nonpolar latitudes for along‐track observations (Zurek & Smrekar, 2007). The specific retrieval version used for this study was v5.2, unless otherwise specified; v5 retrievals incorporate two‐dimensional radiative transfer to account for sharp lateral gradients in temperature and aerosol, leading to improved retrievals in particular at polar latitudes (Kleinböhl et al., 2017). For the 2018 GDS period, v5.3.2 retrievals were used, a reprocessed version containing additional information from other channels on MCS, which for the case of the high dust loadings during the GDS allowed for improved retrievals (Kleinböhl et al., 2020).

## 3 Results

### 3.1 Overview of UVIS Opacity Data Set

The UVIS occultation opacity data set contains aerosol extinction opacities and slant opacities from *L*_S_ = 163° of MY 34 to the end of MY 35, at wavelengths in the entire UVIS range from 200 to 650 nm. Occultations primarily occur at mid‐high latitudes and are sparser in the tropics. Included with the data set are quality control flags for transmission, which decreases toward the surface of the planet.

**Figure 2:**
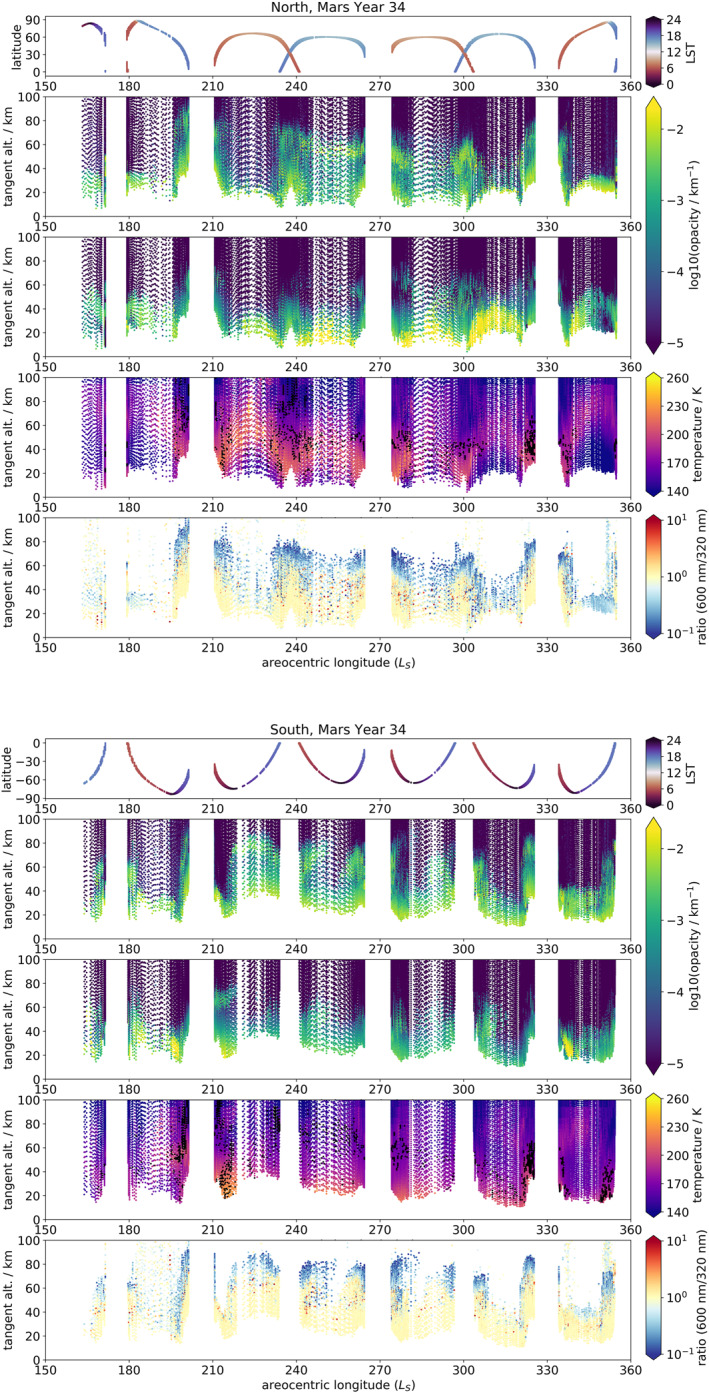
For MY 34 in the northern hemisphere (top five plots) and southern hemisphere (bottom five plots), from top to bottom: UVIS occultation latitude and local solar time distribution; UVIS occultation opacity profiles at 320–360 nm; total (dust + water ice) opacity profiles from the MGCM run with assimilation, matched to the same locations as the UVIS occultations; atmospheric temperatures from the MGCM run with assimilation, matched to the same locations as the UVIS occultations, and overlaid with black dots indicating the approximate location of the hygropause in the MGCM water vapor field, defined here as 70 ppmv (J. Holmes et al., 2021); ratio of UVIS occultation opacities at 600 nm over 320 nm.

**Figure 3:**
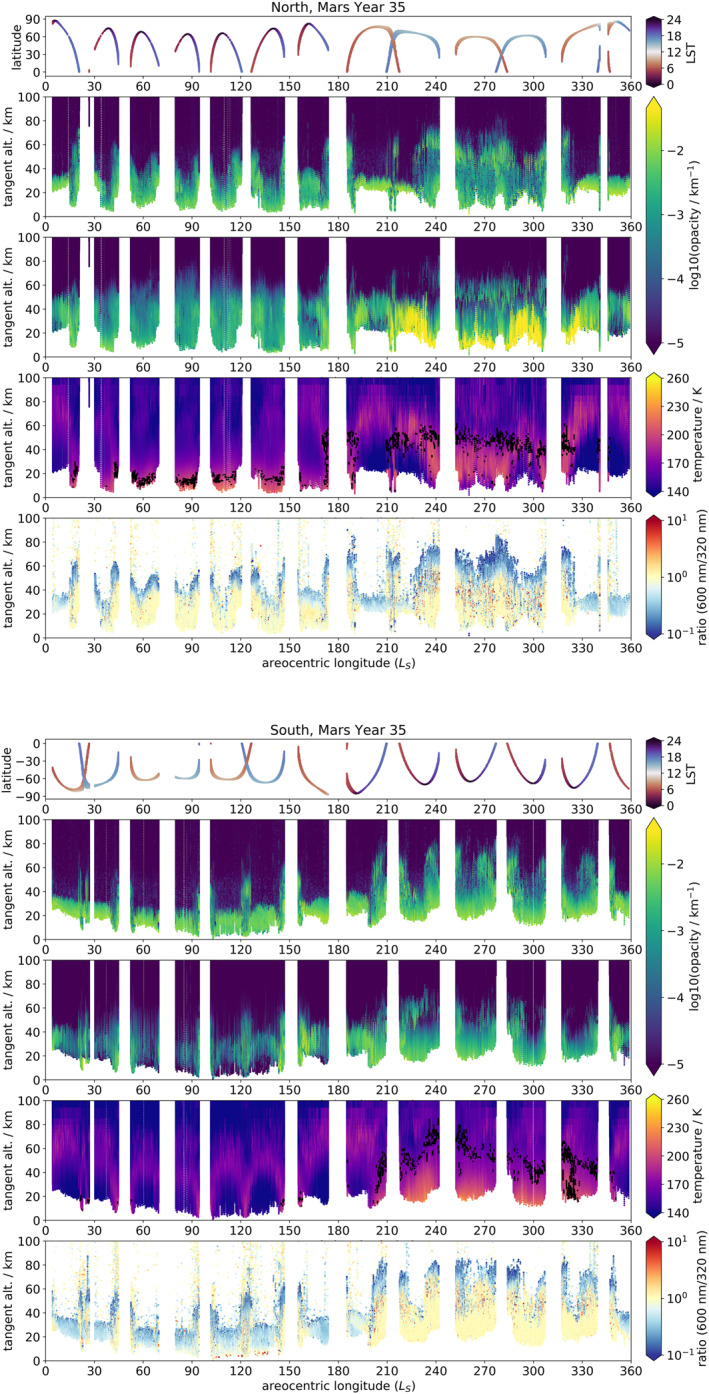
Same as Figure 2 for MY 35.

**Figure 4:**
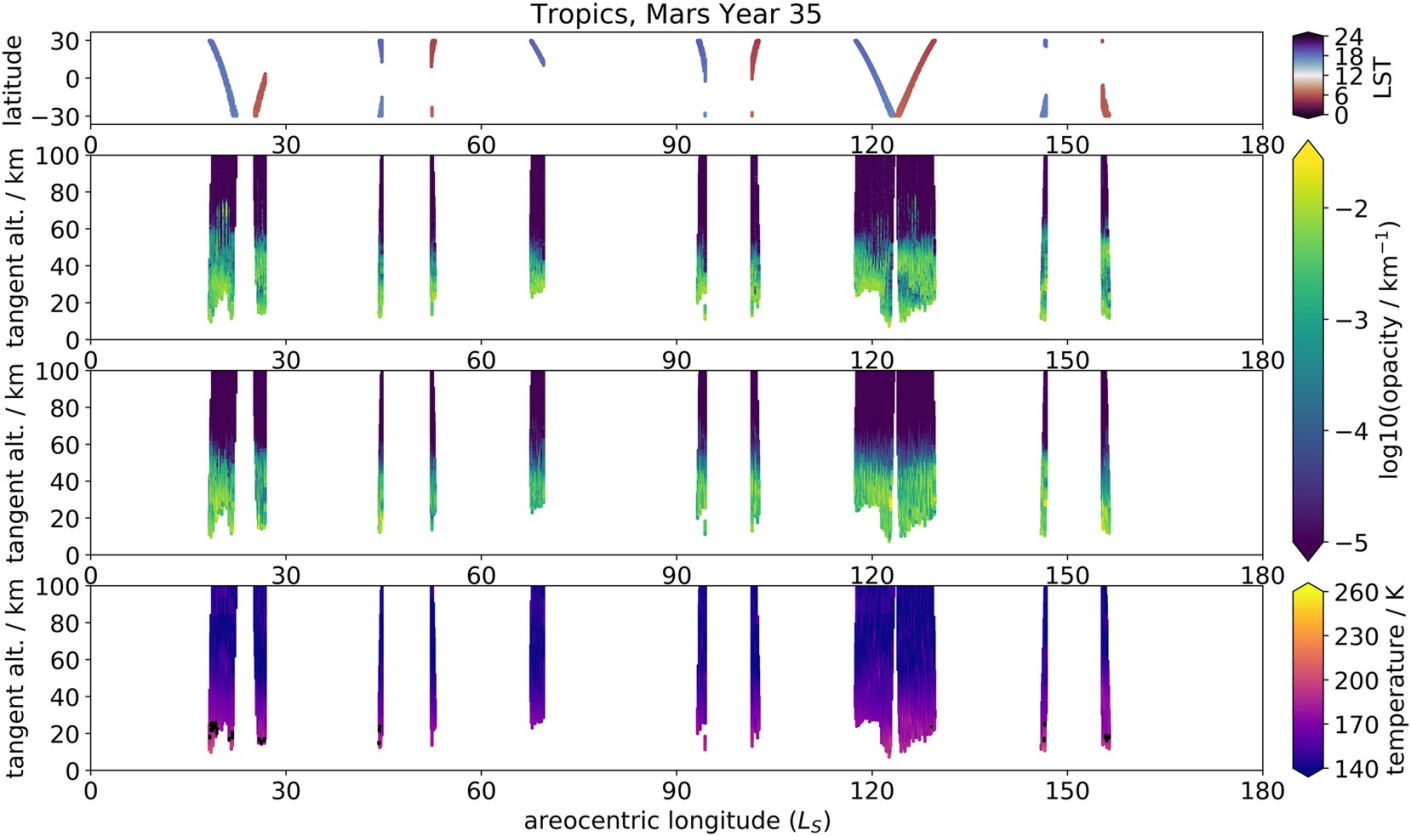
Same as Figure 3, absent the 600/320 ratios and filtered to only include tropical latitudes from *L*_S_ = 0–180°.

Figures 2 and 3 display the UVIS opacity profiles for each hemisphere for MY 34 and MY 35 respectively, averaged between the wavelengths 320–360 nm (sometimes referred to throughout the text as simply 320 nm), together with the total aerosol opacity field in the MGCM with assimilation (obtained by summing dust and water ice opacities at 670 nm) and the temperature field from the same. UVIS profiles were cut off at low altitudes where transmission fell below 1% and above 100 km altitude (where noise begins to dominate), and the MGCM data was masked to match the times and locations of the UVIS profiles. It should be noted that the constantly varying latitude of the observations means that interpretations must be made carefully, as observed changes in the plots could be due to temporal changes, latitudinal changes, or both. This also applies to local solar time of the occultations, which are always at either the sunrise or sunset terminator, though the local solar time of sunrise/sunset can change significantly at higher latitudes; see Figures 2 and 3. Figure 4 shows the same fields (except for the ratios) as presented in Figure 3, but cropped to *L*_S_ = 0–180° and Mars' tropics (defined as 30° S to 30° N), to allow closer examination of tropical opacity features during Mars' aphelion season.

What follows is a brief description of some features of particular note in the UVIS profiles based on Figures 2 and 3; further discussion and interpretation can be found in the next section. The MY 34 profiles cover the period of the MY 34 GDS, which began at *L*_S_ = 185° and had decayed to climatological background dust levels by *L*_S_ = 270° (Kass et al., 2019). The GDS signal is clearly visible in Figure 2 at *L*_S_ = 200°, when opacity levels increase between 30 and 70 km immediately before an extended gap in data due to orbital geometry. After the gap, opacities above 40 km remain high in the northern hemisphere but low in the southern hemisphere, before a persistent high‐altitude high opacity layer appears in the southern hemisphere between 70 and 80 km at *L*_S_ = 215°.

This high‐altitude layer persists until approximately *L*_S_ = 280°, decaying in altitude from up to 80 km to around 40–50 km and present even at the northern and southern latitudes of 60°. Also present are high opacities at lower altitudes, up to 30 km, over roughly the same period. Another high opacity layer is present between 40 and 60 km around *L*_S_ = 290–310°. The MY 34 regional dust storm began at approximately *L*_S_ = 320°, correlating with enhanced opacities in the UVIS profiles up to 80 km. Finally, there was high opacity observed at *L*_S_ = 350–355°up to approximately 60 km.

MY 35 was a martian year without a GDS. The aphelion season (*L*_S_ = 0–180°) is characterized by low opacities above 50 km and high opacity layers at altitudes of 10–50 km. In general there is a low‐level opacity feature throughout this period extending to 20–30 km, and sporadic higher‐altitude detached features at 40–50 km, most notably around *L*_S_ = 90–100° and *L*_S_ = 110–135° in Figure 3. The advent of the dustier perhileion season from around *L*_S_ = 150° brings with it an elevation of the lower atmosphere opacity layer, from 20 km at *L*_S_ = 90° to 30–40 km at *L*_S_ = 190°. High‐altitude opacity layers are visible between 50 and 70 km throughout *L*_S_ = 200–270°. A regional dust storm began at *L*_S_ = 225°, correlating with a high‐altitude opacity layer appearing at 70 km at *L*_S_ = 230° and decaying to 50 km by *L*_S_ = 255°.

The previous subsection briefly described particularly notable opacity features in MY 34 and MY 35 as visible in the UVIS data set. The rest of this paper explores several of these features in greater detail and provides interpretations regarding their nature by way of more hemispherically focused analysis, comparison with the MGCM, and comparison with existing literature.

### 3.2 High‐Altitude Clouds During GDS and Regional Dust Storms in MY 34 and MY 35

The high‐altitude opacity features described during the MY 34 GDS are water ice clouds, as noted in published NOMAD and ACS studies. Both Liuzzi et al. (2020) (NOMAD) and Stcherbinine et al. (2020) (ACS) performed retrievals of water ice for the GDS period, finding a rise in altitude of a water ice cloud layer from 45 to 60 km prior to the GDS to 80–90 km during the height of the GDS at *L*_S_ = 200° which then decayed in altitude to around 50 km as the GDS decayed. Liuzzi et al. (2020) also extended their analysis to the regional dust storm which occurred later in MY 34, beginning at around *L*_S_ = 320°, and found an increase in dust abundance up to 50 km and water ice abundance up to 80 km. The detachment between these two aerosol kinds is visible in Figure 2, which shows a separation between a high opacity layer extending to 50 km and one present at 70–80 km. This high‐altitude layer is explained as the boosted atmospheric transport enhancing water transport toward higher latitudes and thus increasing the height of the hygropause (Heavens et al., 2018; A. Fedorova et al., 2018). This high‐altitude cloud layer was even present at high latitudes, explained dynamically as the enhanced meridional circulation boosting vapor transport toward those latitudes (e.g., Neary et al., 2020). An elegant aspect of the UVIS total opacity data set is that despite its simplicity, it also reveals the features observed during the MY 34 GDS and regional dust storms by retrievals that distinguish aerosol compositions. Comparison with previous such retrievals and the employment of heuristics based on existing research can therefore aid in interpretation of interesting opacity features in data periods which have not yet been subject to retrievals which distinguish between aerosol kinds. This is discussed next in the context of the “A”‐type regional dust storm (Kass et al., 2016) observed in MY 35.

MY 35 experienced an “A”‐type regional dust storm beginning at approximately *L*_S_ = 230° and extending roughly from 60° S to 45° N at its maximum extent. Figure 3 shows that, consistent with the mesospheric water ice clouds observed during the MY 34 regional storm, the MY 35 regional storm inception is correlated with a band of enhanced opacities. In the northern hemisphere, this begins at *L*_S_ = 230° at around 50 km before rapidly rising to 70 km within 5° *L*_S_, then decaying in altitude down to 40 km by *L*_S_ = 270°. In the southern hemisphere however the band remains at 60–70 km even by *L*_S_ = 270°. There is a gap in the southern hemisphere high opacity layer between *L*_S_ = 255–270°, likely related to the high southern latitudes seen by UVIS at this time; this implies the aerosol layer only extended to around 60° S. This asymmetry suggests that the higher atmospheric temperatures and dust loading at this time of year (southern summer) may be responsible for maintaining a mesospheric water ice cloud presence, together with the strong southern summer Hadley cell helping to maintain dust and water aloft and thus keeping temperatures high above the surface. In both hemispheres the high‐altitude water ice cloud presence persists for markedly longer than that associated with the MY 34 regional storm; again, this may have to do with the higher dust content and atmospheric temperatures at this time of year. This water ice cloud presence again indicates a raising of the hygropause, which provides further evidence for the important role of regional‐scale dust storms in enhancing water escape from the martian atmosphere (J. Holmes et al., 2021).

Finally, these high‐altitude water ice cloud features were compared to the MGCM with assimilation to see how well they are reproduced. Interestingly, the southern hemisphere in Figure 2 shows that the MGCM does contain a 70 km water ice feature at high southern latitudes between *L*_S_ = 210–220°of MY 34; a time and location when this is not present in the UVIS data. Throughout the rest of the GDS, there is water ice cloud presence in the MGCM at altitudes up to around 60 km, but not the detached layering at higher altitudes seen in the UVIS data. During the MY 34 regional storm there is some enhanced water ice opacity present around 60–70 km, but of several orders of magnitude less than in the UVIS data. Likewise during the MY 35 regional storm in Figure 3 there is apparent water ice cloud presence at 60 km at *L*_S_ = 255°, but again lower abundance than seen in the UVIS opacities.

By contrast, the distribution of MGCM atmospheric temperature field and approximate hygropause altitude match noticeably better with the UVIS aerosol opacities. The gradual decay in atmospheric temperatures during the GDS from *L*_S_ = 220–300° correlates well with the gradual decay in the height of the mesospheric water ice cloud layer in the UVIS data over the same period; the same is the case for the MY 35 regional storm. Likewise, the spike in atmospheric temperatures following the onset of the MY 34 regional storm at *L*_S_ = 320° aligns well with the high‐altitude water ice cloud layer present in the UVIS data in the same time. As temperatures in the MGCM are calculated by assimilating MCS temperature profiles, this suggests that the failure of the MGCM to replicate the mesospheric water ice may be due to a lack of mesospheric water vapor or CCN, or limitations of the cloud parametrization. However, the approximate hygropause altitude as calculated from the MGCM accords well with the location of the high‐altitude cloud layers in the UVIS data, which indicates that the water vapor content in the mesosphere is being reasonably well reproduced by the assimilation. The raising of the hygropause during intense GDS dust loading also agrees with previous observations of the MY 28 GDS (Heavens et al., 2018; A. Fedorova et al., 2018).

As a final note, opacities at the bottom of the datasets are significantly (at least an order of magnitude) higher in the MGCM than in the UVIS data set during the GDS and after, namely from *L*_S_ = 210–320° of MY 34. As this is most likely dust at these altitudes (Liuzzi et al., 2020), this would imply that the MGCM is overestimating dust presence at roughly 10–30 km, meaning that (assuming the CDOD value is correct) more of the atmospheric dust within the column is located in the bottom scale height or two of the atmosphere than represented in the MGCM; or else, the CDOD value is being overestimated, possibly from spurious opacities due to water ice cloud presence. This has implications for future representation of vertical dust structure in the MGCM, which may need to be adjusted to show a greater dust presence in the bottom scale height of the atmosphere. Future nadir retrievals of CDOD from NOMAD, in conjunction with MCS and NOMAD profiles, should help to better constrain the atmospheric dust content in this crucial bottom 10 km.

### 3.3 Aphelion Clouds in MY 35

The MY 35 aphelion season is characterized by the presence of high opacity features between 10 and 50 km in the UVIS opacity profiles, increasing in altitude toward the tropics (Figures 3 and 4). These features tend to occur at higher altitudes in the northern than the southern hemisphere. The omnipresent high opacity layers below 30 km in both hemispheres at this time most likely correspond to combined water ice from the ACB and dust, the former having been observed to extend from 17 to 45 km and the latter up to 20–30 km at this time of year (Smith et al., 2013). The greater altitudes of high opacity features in the north also accord with the northern tropical ACB having been observed to occur at higher altitudes than in the south (Smith et al., 2013), as well as the fact that the north is the spring/summer hemisphere at this time of year, with resulting greater dust activity. There is also a visible slope in the altitude of the background haze layer in the northern hemisphere, increasing in altitude as the ground track of the latitude approaches the equator; for example, at around *L*_S_ = 45°, *L*_S_ = 75°, *L*_S_ = 100°, and *L*_S_ = 120°. The latter of these has a detached structure in the south; this is discussed further below.

As well as the omnipresent haze below 30 km, there are some features visible in the UVIS opacities extending up to ∼50 km altitude. These are more prevalent nearer the tropics, and visible in both hemispheres. One especially noticeable such feature occurs in both hemispheres around *L*_S_ = 120° and extends up to 50 km. It appears to be detached in the southern hemisphere, but an extension of the background haze in the northern. The particular visibility of this specific feature may be due to the fact that UVIS occultations occur very close to the equator itself at this time, while they are generally constrained poleward of 30° over this aphelion period. The altitude range of this feature is consistent with the higher altitude range of the ACB (Smith et al., 2013) and is higher than the generally reported altitude range (15–25 km) of detached dust layers (e.g., Heavens et al., 2011a). The MGCM local temperature minimum at 50 km/maximum below 50 km at *L*_S_ = 200° also suggests the presence of cooling (higher albedo) water ice over warming (lower albedo) dust; this temperature structure is particularly visible in the southern hemisphere. However, some detached dust layers have been observed at extremely high altitudes over regions of high topography, for example, at 55 km (Clancy et al., 2019) and even up to 75 km with a large (<1,000 km) spatial footprint (Heavens et al., 2015), likely connected to topographic forcing (Heavens et al., 2015). This particular feature should therefore be of interest for more detailed future examination.

There are also some lower‐opacity features during the aphelion season worthy of noting, as they correlate with lower MGCM temperatures at ∼50 km, possibly indicating water ice presence. One such example occurs in both hemispheres around *L*_S_ = 40–50°, on either side of the gap in UVIS data. These features extend up to ∼50 km and correspond to tropical latitudes, consistent with the ACB. There is also a slight increase in the altitude of the modeled northern hemisphere hygropause, suggesting higher water content in the atmosphere. Another such example can be found in both hemispheres around *L*_S_ = 140°, shortly before the UVIS data gap and again at tropical latitudes up to ∼60 km. There also appears to be a corresponding temperature minimum above this feature in both hemispheres, and a slightly elevated northern hygropause.

The MGCM aerosol opacity field generally shows good agreement with the UVIS opacities, capturing the gradual dip in altitude of the sub‐30 km opacity layer from *L*_S_ = 0–90° and then its gradual rise again from *L*_S_ = 90–180°. The MGCM also aptly reproduces the high‐altitude opacity feature in the southern hemisphere at *L*_S_ = 120°. However, particularly in the northern hemisphere, the MGCM does appear to overestimate opacities above 50 km and underestimate them below, portraying more of a uniform haze than the sharp structure in the UVIS opacities. This suggests that while the cloud scheme is ably reproducing general seasonal and latitudinal trends, there is room for improvement in representing the vertical structure of the ACB.

### 3.4 High‐Altitude Clouds During Non‐Regional Dust Periods of MY 35 Perihelion

The mesospheric water ice features present during global and regional dust storms have already been discussed, but the UVIS opacities for MY 35 also show similar features during the perihelion season before the beginning of the MY 35 A‐type regional storm at *L*_S_ = 230°. These features can be seen at mid‐high latitudes in both hemispheres around *L*_S_ = 210°, at approximately 50–80 km (Figure 3). The northern feature is significantly shorter‐lived and at a lower altitude (50 km), while the southern feature extends from *L*_S_ = 200–220° and 50–80 km. The former appears between approximately 45‐70° N and the latter between approximately 30‐60° S.

The altitudes of these features are greater than those observed for polar hood water ice clouds (Benson et al., 2010, 2011), but along with their latitudes and season agree well with perihelion mesospheric water ice clouds as detected by Clancy et al. (2019) with CRISM in previous martian years. Clancy et al. (2019) noted a sharp dichotomy in mesospheric (50 km+) water ice presence between the colder aphelion season and the warmer perihelion season, with the vast majority of water ice above 60 km being detected between *L*_S_ = 160–360°. This perihelion mesospheric water ice showed some clustering around mid‐high latitudes in both hemispheres, which is also consistent with the UVIS opacities, though there is an intrinsic bias in these due to the lower frequency of equatorial occultations. By contrast, mesospheric CO_2_ ice clouds detected by Clancy et al. (2019) occurred primarily during the colder aphelion season, and at tropical latitudes, consistent with many previous studies of mesospheric CO_2_ ice clouds (e.g., Aoki et al., 2018; Määttänen et al., 2010; McConnochie et al., 2010; Vincendon et al., 2011). Interestingly, McConnochie et al. (2010) also identified mesospheric cloud features at twilight at mid‐latitudes during the perihelion season, but were unable to conclusively classify their composition. Drawing from these previous results, therefore, the most consistent explanation for the mesospheric opacity features observed in UVIS data is the presence of water ice clouds.

Finally, the UVIS opacities were compared to the MGCM opacities and temperatures. The detached opacity structures are not reproduced by the MGCM, but the temperature and hygropause structure show supporting evidence that these opacity layers are water ice. In both hemispheres, the approximate altitude of the hygropause roughly doubles (from 20 to 40 km) from the aphelion season to the perihelion season, boosted by the generally higher atmospheric temperatures. In the southern hemisphere, where this opacity layer is greater in spatial and temporal extent, the hygropause rises from below 20 km to 40–60 km between *L*_S_ = 200–220° and as occultation latitudes move from 70° S toward the tropics. This matches well with the altitude of the UVIS opacity layers in the southern hemisphere, indicating availability of water for condensation. Likewise in the north, a 40–50 km hygropause in the MGCM is present at the time and location of the northern opacity layer at 50 km.

### 3.5 A Brief Note Regarding CO_2_ Ice

As mentioned previously, there have been numerous confirmed detections of CO_2_ ice clouds in the martian mesosphere, the majority of which have been at equatorial latitudes during the colder aphelion season (e.g., Aoki et al., 2018; Määttänen et al., 2010; McConnochie et al., 2010; Vincendon et al., 2011), with typically observed altitudes of 55–80 km above the dayside tropics (Clancy et al., 2007, 2019). The theorised dynamical origins of such clouds from the creation of cold pockets by gravity waves and/or thermal tides (e.g., González‐Galindo et al., 2011; Spiga et al., 2012) may be related to the calculated short lifespans of such features, which could be as low as minutes (Listowski et al., 2014). The equatorial location and ephemerality of mesospheric CO_2_ clouds intrinsically makes identification of such features difficult here, as UVIS occultations are relatively sparse at tropical latitudes and the annual‐scale *L*_S_‐altitude plots presented here are intended to display spatially and temporally extended features on the order of at least several degrees *L*_S_ and several kilometres in depth. Figure 3 shows no significant detached high opacity features above 40–50 km in either hemisphere during the aphelion season, with the possible exception of a small feature visible in the north at *L*_S_ = 15–20°near the equator at approximately 70 km, which does correlate to a high‐altitude temperature minimum in the MGCM. As stated though, the sparse nature of mesospheric CO_2_ ice clouds, and their reliance on fine‐scale and longitudinally variable dynamics, makes it difficult to draw inferences in a broad study such as this one. We therefore do not draw conclusions regarding the possible presence of mesospheric CO_2_ clouds in the UVIS opacity data set and leave this to future studies, which may benefit from the dusk/dawn local times of the occultations for better understanding of the nature of these phenomena.

### 3.6 Aerosol Particle Size and Composition

Figures 2 and 3 also contain panels for each hemisphere and MY showing the ratio of the aerosol opacity at 600 nm compared to that at 320 nm. 600 nm was chosen as a representative comparison value, but ratio plots of 500 and 550 nm (not shown) show a very similar pattern. Ratios were only plotted when both the 320 and 600 nm opacities were >10^−5^, to eliminate noise at low opacities. For times and locations with high opacities, there is generally good agreement between the 320 and 600 nm values (i.e., the ratio is ∼1), for example, during the GDS and RDS periods of MY 34, and below 40 km during MY 35 (especially in the perihelion season). Above the high opacity features, however, there is a consistent band of ratios with value < 1, implying lower opacity at 600 nm than at 320 nm. Such values are consistent with attenuation of the signal at shorter wavelengths as the occultation passes through the atmosphere however (an example can be seen in Patel, Sellers, et al., 2021, Figure 5), and do not necessarily imply compositional or particle size information. There are some potential features of interest where there is heterogeneity in the value of the ratio within areas of high opacity, for example, between *L*_S_ = 255–310°of MY 35 in the northern hemisphere. This could suggest a corresponding heterogeneity in particle sizes or composition. However a full analysis is beyond the scope of the techniques used in and appropriate for this study.

The so‐called Angstrom Coefficient technique (e.g., Gröller et al., 2018; Määttänen et al., 2013) can be used under certain conditions, whereby the spectral slope of opacity can indicate changes in particle size and/or composition throughout the atmosphere. Unfortunately, such a technique is not suitable for the retrievals presented in this work. We employ a direct opacity analysis, rather than a forward‐modeling technique which would be appropriate for an Angstrom Coefficient analysis. In addition, the Angstrom Coefficient technique is only suitable for a short wavelength range where there is a gentle curve in the transmission, rather than the long wavelength ranges employed here. The 320–360 nm band is used in this analysis as a corollary of the preceding ozone analysis, from which these aerosol opacity retrievals are derived (Patel, Sellers, et al., 2021). There is some further discussion of this issue in the Supplementary Information, including a comparison of the opacity at 450 nm to investigate any broad changes in the transmission signal between 320 and 600 nm. A detailed analysis using UVIS data of possible particle size or compositional changes of aerosol is left to future work using more appropriate retrieval techniques.

## 4 Conclusions

This article has presented a new data set freely available to the martian atmospheric community, consisting of total extinction opacity profiles derived from solar occultations by the ExoMars NOMAD/UVIS instrument for the 320–360 nm spectral range. The data set contains opacity profiles for the period covering the MY 35 Global Dust Storm and regional dust storm, and the entirety of MY 35, allowing investigation of both the extreme dust loading of a GDS year and of a full typical martian year. Solar occultations occur at local dusk and dawn, allowing research into the vertical opacity structure of Mars' atmosphere at the terminator where interesting day‐night transition processes may be occurring.

Key features of interest in the UVIS opacity data at the 320–360 nm range have been identified, interpreted according to context and previous literature, and compared to an MGCM with data assimilation. These include the mid‐high latitude mesospheric water ice clouds associated with the high dust loading of the MY 34 GDS and regional dust storm, the seasonally recurring aphelion cloud belt, and perihelion cloud layers in MY 35 – some associated with regional dust storms, others not. Existing retrievals of aerosols in MY 34 allowed identification of the high opacity layers observed in this study above the high dust loading as water ice, and this enabled the inference of very similar layers above regional dust storms in MY 35 as water ice as well. This analysis validates the UVIS opacity data set as reproducing the observed mesospheric cloud structure in MY 34 and shows its use for ongoing analysis as UVIS continues to perform solar occultations of the atmosphere. With this simple but extensive data set combined with reasonable inference, it has been shown that mesospheric water ice cloud is regularly seen above regional‐scale dust events, and that the season of such events is linked to the temporal extent of these cloud layers. Earlier storms, like the MY 34 GDS (*L*_S_ = 185°) and MY 35 A‐type storm (*L*_S_ = 230°), are associated with longer‐lived mesospheric cloud layers than later storms like the MY 34 regional dust storm (*L*_S_ = 320°) and the MY 35 C‐type storm (*L*_S_ = 320°). This implies an important role for regional dust storms in martian atmospheric escape.

MGCMs and data assimilation also provide an important function: on the one hand, they can aid in interpretation of specific opacity features via (e.g.,) comparison of the temperature structure; on the other, the UVIS opacity data set is a potential tool for validation of MGCM aerosol representation, specifically vertical structure. The utility of assimilating temperatures in particular is that it enables better representation of the real thermal structure and dynamics of the martian atmosphere. This provides a valuable interpretative tool for the UVIS opacities, for example, by showing how enhanced lower atmospheric temperatures and a raised hygropause correlate with high‐altitude detached opacity layers in the UVIS data, providing supporting evidence for their interpretation as water ice. In the other direction, it is clear from this analysis that current water ice parametrizations struggle to reproduce perihelion mesospheric clouds. Even during the ACB, when the MGCM shows good agreement with the UVIS opacities, the observations suggest a more vertically confined and sharper aerosol structure than shown in the MGCM. The UVIS data set therefore offers a good opportunity for validation of aerosol vertical structure in MGCMs simulating both dust and water ice, and development of improved parametrizations.

## Supporting information

Supporting Information S1

## Data Availability

Data used in this paper is freely available. UVIS opacity occultation profiles can be found in M. Patel, Sellers, et al. (2021), and the comparison assimilation MGCM data masked to match the times/locations of the UVIS profiles can be found in P. Streeter et al. (2021).
