# Supplementary material for: Vertical Aerosol Distribution and Mesospheric Clouds From ExoMars UVIS
Source: J Geophys Res Planets. 2022 Apr 27;127(5):e2021JE007065. doi: 10.1029/2021JE007065 (PMC9286791; doi:10.1029/2021JE007065)
Supplement: Supplementary file 1 — Supporting Information S1 [file JGRE-127-0-s001.pdf]

Supporting Information for "Vertical aerosol distribution and mesospheric clouds from ExoMars UVIS"

Paul M. Streeter[1], Graham Sellers[1], Michael J. Wolff[2], Jonathon P. Mason[1], Manish R. Patel[1,2], Stephen R. Lewis[1], James A. Holmes[1], Frank Daerden[4], Ian R. Thomas[4], Bojan Ristic[4], Yannick Willame[4], Cédric Depiesse[4], Ann Carine Vandaele[4], Giancarlo Bellucci[5], and José Juan López-Moreno[6].

[1]School of Physical Sciences, The Open University, Walton Hall, Milton Keynes, UK

[2]Space Science Institute, Boulder, Colorado, USA

[3]Space Science and Technology Department, Science and Technology Facilities Council, Rutherford Appleton Laboratory, Oxfordshire, UK

[4]Royal Belgian Institute for Space Aeronomy (IASB-BIRA), Brussels, Belgium

[5]Istituto di Astrofisica e Planetologia Spaziali (IAPS/INAF), Rome, Italy

[6]Instituto de Astrofisica de Andalucía (IAA), Consejo Superior de Investigaciones Científicas (CSIC), Granada, Spain

Contents of this file

1. Text S1
2. Figures S1 to S2

Introduction

This Supporting Information contains additional figures to provide additional context for the results provided in the manuscript. Figures S1 and S2 are described in Text S1,

and complement the discussion in the "Aerosol particle size and composition" section of the main manuscript.

Text S1.

Figs. S1 and S2 show the latitude-LST distribution of UVIS occultations, ratio of 600 nm to 320 nm opacities, and 320 nm opacity values as shown in the main text. They also include additional panels showing a normalised value of the difference between the "expected" value of the aerosol opacity at 450 nm, assuming a linear gradient in transmission between 320 nm and 600 nm, and the actual retrieved value of the aerosol opacity at 450 nm. This is labelled "nonlinearity index" in the panels. This value is intended to capture non-linear behaviour in the transmission at 450 nm, which could indicate the presence of changes in aerosol particle size and/or composition. Red values indicate that the "expected" opacity at 450 nm is larger than the retrieved opacity at 450 nm, and blue the opposite.

The red banding in the nonlinearity index above the high-opacity layers in both MY correlates well with where the ratio of 600 nm to 320 nm opacity is <1. This agrees well with the notion suggested in the main article text that the <1 ratios in these locations are due to attenuation of the signal towards shorter wavelengths, as such an attenuation would be reflected in the transmission as a steeper-than-linear drop in transmission towards shorter wavelengths; hence a higher "expected" than retrieved opacity at 450 nm. This does not necessarily suggest any meaningful changes in particle size and/or opacity.

As stated in the main text in reference to the 600/320 nm ratio values, possible features of interest are identified when opacities are high. The most notable is between L_S=255-310°in MY 35, in the northern hemisphere below ∼50 km. Here, there is high heterogeneity

in the value of the nonlinearity index, even despite the fact that this is a region of high opacity where normally there is good agreement in the 320 nm and 600 nm opacities. This region could therefore provide a good starting point for a future detailed analysis of aerosol particle size and composition from UVIS occultation data. As stated in the main text, such an analysis is beyond the scope and techniques of this study.

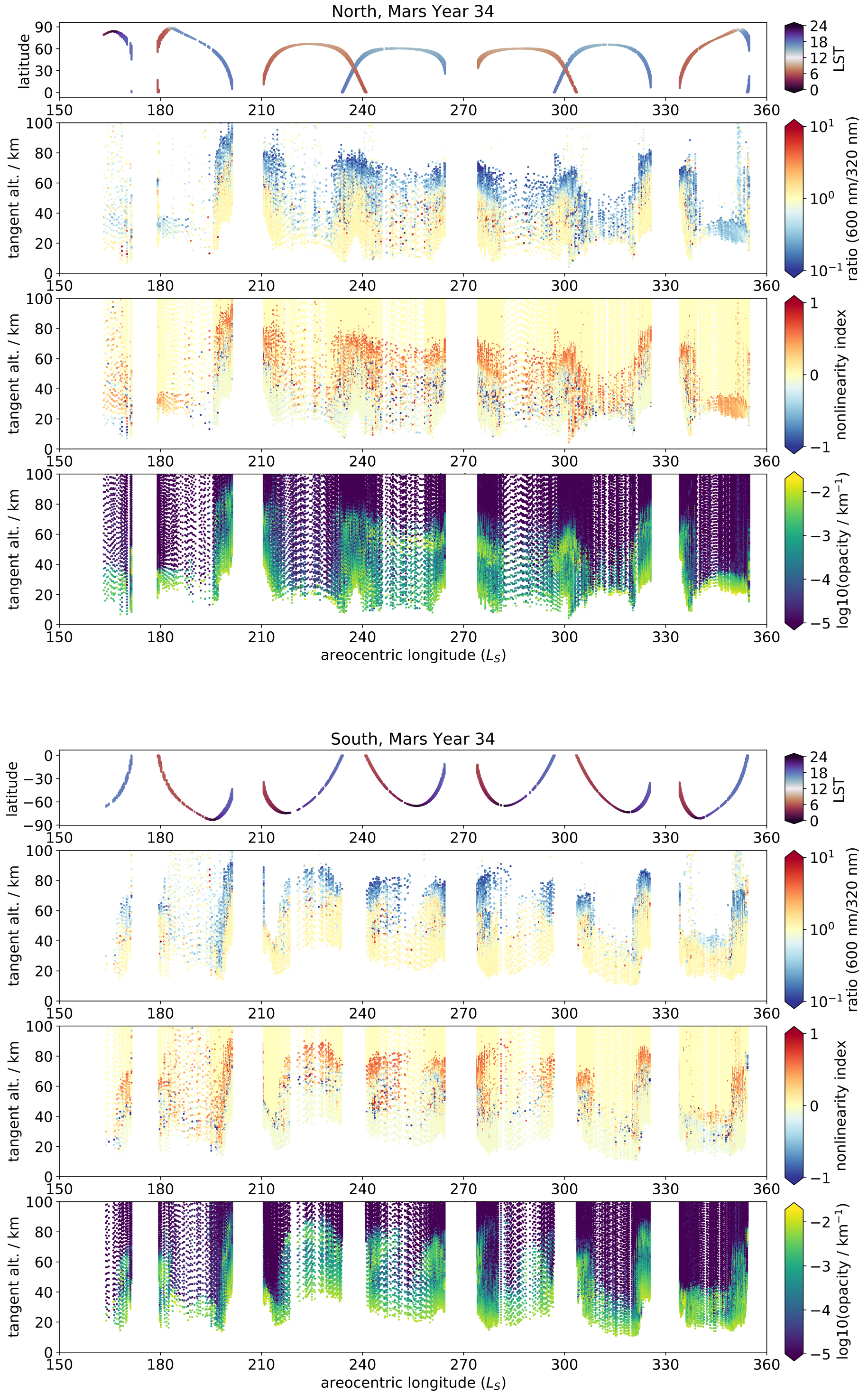

Figure S1. For MY 34 in the northern hemisphere (top four plots) and southern hemisphere (bottom four plots), from top to bottom: UVIS occultation latitude and local solar time distribution; ratio of UVIS occultation opacity profiles at 600 nm over 320 nm; "nonlinearity index", defined as the difference between the expected value of UVIS occultation opacities at 450 nm assuming a linear gradient in transmission between 320–600 nm, and the actual value of UVIS occultation opacities at 450 nm, then normalised to the expected value; UVIS occultation opacity profiles at 320–360 nm.

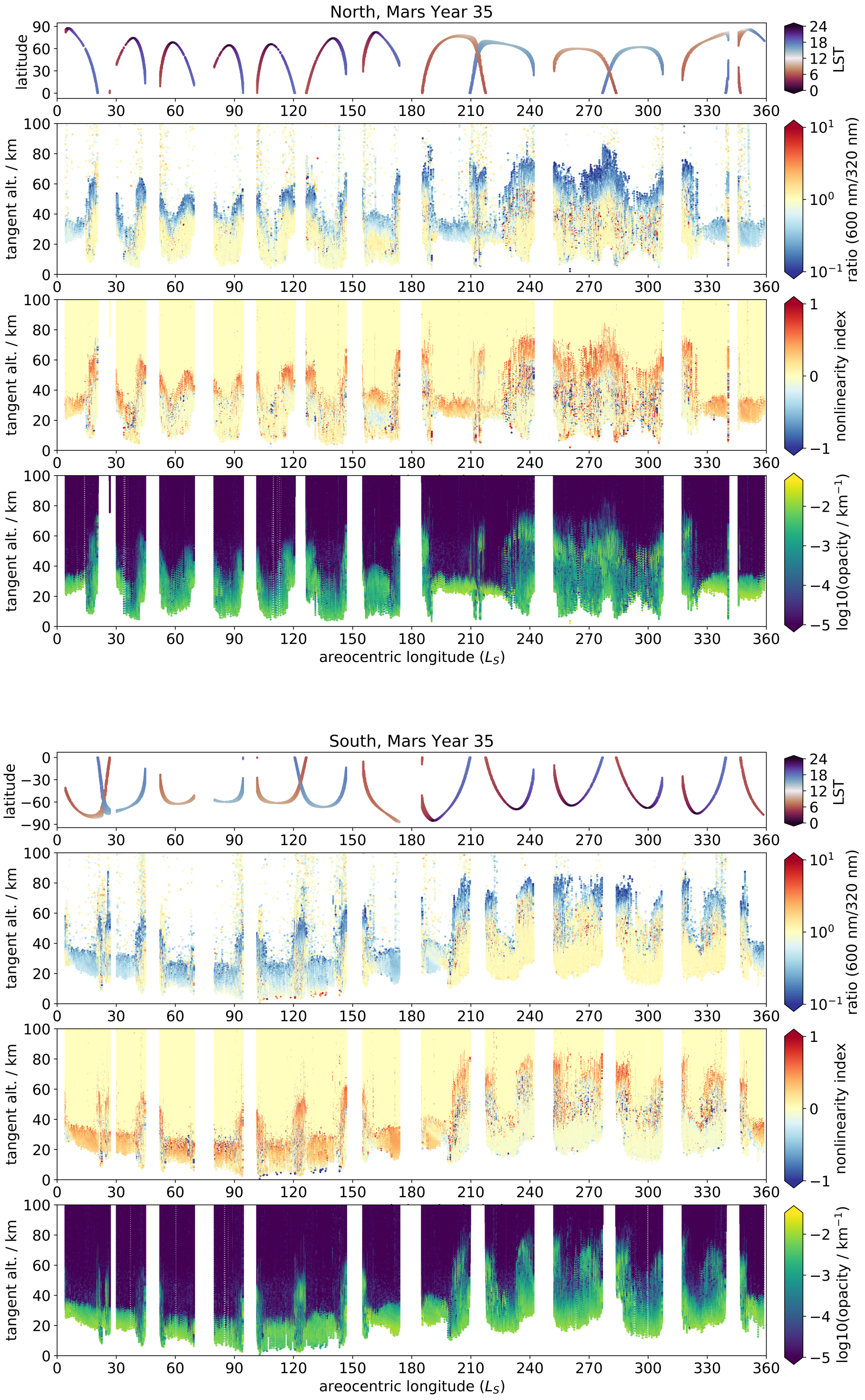

Figure S2. Same as Fig. S1 for MY 35.
